# Supplementary material for: Are carbon emissions trading and green financial instruments synergistic? -Comprehensive quantitative research based on content analysis
Source: PLoS One. 2024 Mar 7;19(3):e0298601. doi: 10.1371/journal.pone.0298601 (PMC10919601; doi:10.1371/journal.pone.0298601)
Supplement: S1 Data — (ZIP) [file pone.0298601.s001.zip › Data/地级市Z_F工作报告.zip!/░▓╗╒/╗┤▒▒╩╨/└┤╫╘╣1⁄2╓┌║┼í╛┬φ┐╦╩2╛▌═°í┐_2017.docx]

各位代表：

现在，我代表市人民政府，向大会报告政府工作，请予审议，并请市政协委员和其他列席人员提出意见。

2016年：知难而进，“十三五”实现平稳开局

刚刚过去的一年，是“十三五”的开局之年，也是我市应对各种风险挑战，探索转型发展新路的一年。一年来，在市委的坚强领导下，在市人大、市政协的监督支持下，市政府团结带领全市人民，坚持稳中求进工作总基调，以供给侧结构性改革为主抓手，统筹推进稳增长、调结构、惠民生、防风险各项工作，经济发展稳中有升，社会大局和谐稳定。预计全年生产总值780亿元，比上年增长5%。固定资产投资963亿元，增长4%。财政收入93.3亿元，较上年略有增长；财政支出142.5亿元，较上年增加11.1亿元。社会消费品零售总额315.1亿元，增长11.2%。外贸进出口总额6亿美元，增长5%。城镇、农村居民可支配收入27230元、10570元，分别增长6%、7%。城镇新增就业超过全年目标。节能减排完成省定任务。

一年来，主要做了以下工作：

一是扎实推进“去降补”，经济实现平稳增长。制定供给侧结构性改革总体方案和配套政策措施，推动改革任务落地生根。六大行业全面回升，省属企业扭亏增盈，县区工业提质增效。去产能方面，压减煤炭过剩产能489万吨，妥善分流安置职工9789人，顺利通过省和国家级勘察验收。全力支持企业脱困转型发展，累计为省属煤炭企业协调融资189亿元。去库存方面，以满足新市民住房需求为重点，出台鼓励农民进城购房落户优惠政策。全市房地产库存面积同比下降21%，其中商品住宅去化周期8个月，处于合理区间。降成本方面，全面落实降成本“21条”措施，打出“组合拳”。规模以上工业企业百元主营业务收入成本同比下降1.6%。预计减免抵退各项税费10亿元，企业缓缴社保费11.2亿元。新增电力直接交易用户9家，年节约企业购电成本1.6亿元。工商业电价实现“三连降”。开展“干部帮联企业”“百名金融顾问服务百家企业”等系列活动，为企业解决实际问题239个，发放续贷过桥资金18.1亿元。补短板方面，累计完成基础设施建设投资104.4亿元，增长80.6%。淮萧客车联络线工程主体完成，符夹线扩能改造工程稳步推进，淮北—合肥、淮北—阜阳城际铁路项目纳入皖北城际铁路网规划，徐州—淮北—阜阳高速公路项目列入省级规划。组织实施PPP项目6个、总投资57亿元，其中淮水北调市级配水工程、中湖地质环境治理、梧桐中路改造入选全国第三批PPP示范项目。南坪船闸和浍河航道疏浚工程开工建设。S101合相路一期建成通车，S203煤化工快速通道全线贯通，主城区与南部次中心的交通联系更加紧密。

二是全面实施“调转促”，转型升级步伐加快。全市战略性新兴产业实现产值280亿元，占规模以上工业比重达15.4%。规模以上工业企业总数达838家。规模以上工业增加值460亿元，增长3.5%。加强产业升级和技术改造，完成技改投资192亿元，新增“安徽工业精品”4个，安徽矿机电液控液压支架入选省重大技术装备首台（套）名单，陶铝新材料推广应用得到省委、省政府大力支持。召开企业家创新创业大会，支持民营经济发展，民营经济增加值占生产总值比重达57%。传化公路港物流、凤凰山邮政快递物流园投入运营。濉溪县获批全国电子商务进农村综合示范县。隋唐运河古镇“五凤三阁”建成开放，并获批省影视制作园区。四季榴园获批国家4A级旅游景区、南湖景区通过专家评审。相山区、烈山区入选第二批国家全域旅游示范区创建名单。全年粮食总产126.9万吨，实现“十三连丰”，专用品牌粮食建设走在全省前列。家庭农场、农民合作社分别发展到2145个、1294个，规模以上农产品加工企业达213家。“四新”经济加快成长，“双创”活力加速释放，“源创客”“梧桐树”“方正智谷”等众创空间建成运营。果力科技“柔性智能机械手”获首届中国“双创”机器人创客大赛第一名。新认定国家高新技术企业21家，全市高新技术产业增加值114亿元，占规模以上工业比重达24.8%。万人发明专利拥有量达4.23件。培育中国驰名商标5件，“淮北石榴”成为我市首个中国地理标志商标。安徽省铝制品质量监督检验中心建成使用，中国芳香产业技术创新战略联盟落户相山，杜集高端装备制造基地被认定为国家火炬特色产业基地。濉溪县位列全国县域经济投资潜力百强县（市）第49位。

三是强力推进招商引资，开放合作持续深化。建立“4664”招商工作机制，坚持市级领导带头招商、招商团重点招商，招商项目数量和质量显著提升，全年新签约项目322个，新开工项目271个，其中新开工5亿元以上项目20个。12个重大招商项目顺利推进，相山海螺水泥、企成电气、瑞柯玛锂离子动力电池、台湾文创园等一批项目签约或开工建设，完美食品、南翔云集文化特色街区等一批项目成功引进。产业招商取得新突破，煤化工基地焦化二期、卓泰一期建成投产，填补我市“有焦无化”的空白；金龙机电二期、京信电子等企业落户，壮大电子信息产业；万达城市综合体、中农批物流园开工建设，有力带动现代服务业提档升级。节会招商取得新进展，成功举办2016淮北食品工业博览会、淮北煤基新材料及化学品发展论坛、石榴文化旅游节等盛会，提升了城市知名度和影响力。加快园区转型升级，开展国家新一轮开发区审核公告目录修订工作，清理盘活闲置厂房70余万平方米，以“二次招商”推动“腾笼换鸟”。积极培育外贸主体，新增进出口实绩企业18家。青龙山铁路无水港基本建成，海聚科技公用型保税仓库建成运营，贸易便利化水平明显提升，促进我市外向型经济快速发展。

四是统筹推进城乡建设，城市功能更加完善。组织新一轮城市总体规划修编，编制完成城市特色风貌规划、海绵城市建设规划、城市管廊规划、省级开发区总体规划及控制性规划。获批国家低碳城市、国家气候适应型城市建设试点。结合采煤塌陷实际，争取国土部核减耕地保有量面积20.9万亩、基本农田面积12.9万亩，为城市建设提供有力保障。把文明城市创建与城乡建设相结合，鹰山南路、长山南路铁路立交桥等10个项目建成通车，东部新城、高铁新区11条路网加快推进，惠苑路步行街、环山东路西段等5条“断头路”顺利贯通。新建雨水管涵14公里，改造雨水泵站8座，有效应对夏季两轮特大暴雨袭击。完成黑臭水体调查摸底，建成污水管网三期工程15.3公里，实施长山南路绿带等10条道路绿化、黎苑公园等14个游园节点提升工程，新建绿道36公里，造林绿化面积2.9万亩，东湖宜园建成并向市民开放，生态城市展露迷人新姿。新铺设供水主干管网60.5公里，新建、改造燃气管网85公里，增开107路、109路等公交线路6条，城乡基础设施一体化加快推进。开展相山路等城市管理示范路创建活动，餐厨垃圾处理厂建成试运行。推动棚户区改造与老旧小区整治、公共设施完善相结合，实施棚改项目90个、改造4.8万套，货币化安置率达87.4%。相山公园二号门停车场等一批便民设施建成使用，拓展了城市公共活动空间。美丽乡村建设全面铺开，完成农村道路畅通工程511公里、改造农村危房900户，解决了2.3万人饮水安全问题，316个自然村同步启动村庄环境整治。烈山区美丽乡村标准化试点通过国家验收，朔里镇入选省首批特色小镇。智慧城市建设成效显著，引进华为科技、北大方正、科大讯飞等行业领军企业，在全国首创以PPP模式建设智慧城市。社会服务管理信息化平台覆盖全部城区和276个农村居委会，下沉服务事项居全省首位，“爱·淮北”门户网站和APP上线运行，变群众跑腿为数据跑路，共享指尖上的智慧生活。

五是深化重点领域改革，发展活力显著增强。持续深化“放管服”改革，市级行政审批项目精简到151项，精简率达61%。在全省率先全面启用政府权力清单网上运行平台，市县两级13013项涉权事项全部上网运行，行政权力网上运行监管与编制实名制管理实现互联。建立公共服务清单和行政权力中介服务清单，形成“3+2”新型清单制度体系。推进商事制度改革，在全省较早实行“五证合一、一照一码”登记制度，新增私营企业4141户、个体工商户1.2万户，分别增长26.6%、14.6%。开展市直行政事业单位国有资产清查，摸清“家底”，盘活市工业学校原校区等5处闲置资产。市建投集团持续做大做强，县区融资平台改革全面启动。设立徽银城镇化、安元、天使等一批基金，首次发行15亿元小微企业增信集合债。全省唯一的股权托管交易中心皖北融资中心建成运营，38家企业在省区域四板、3家企业在“新三板”挂牌。创新开展税融通、“4321”新型政银担等业务，全市新增贷款、直接融资双双突破百亿元。机关事业单位工作人员养老保险制度改革全面实施。公务用车制度改革走在全省前列。成立市城市管理工作委员会，调整完善市、区规划建设管理体制，事权财权和人员编制划转实现平稳过渡。农村土地确权登记颁证工作基本完成，颁发土地确权证书31.3万张。杜集区双楼村成为我市首个集体经济“股份村”。我市获批创建国家社会信用体系建设示范城市。

六是着力保障改善民生，社会大局和谐稳定。34项民生工程全面完成，民生工作连续9年稳居全省第一方阵。扎实推进脱贫攻坚，22个贫困村结对帮扶实现全覆盖，全年4个贫困村出列、18790人脱贫。企业退休人员基本养老金实现“十二连涨”，城乡低保标准实现“六连增”，提前实现农村低保标准和扶贫标准“两线合一”。超额完成学前教育三年行动计划第二期任务。淮北技师学院正式挂牌，“相城金蓝领”万人计划启动实施。市博物馆入选首批中国华侨国际文化交流基地。综合医改深入推进，市县区计划生育年度考核全面实现争先进位，相山区荣获全国计划生育优质服务先进单位称号。新型农村合作医疗工作领跑全省。上榜“中国好人”127位，名列全省第一、全国前列，连续五届蝉联全国无偿献血先进城市。平安淮北建设成效明显，综治维稳信访信息系统和基层工作中心（站）全面建成，群众安全感和满意度指数保持皖北首位。探索司法破产和解新路径，成功化解房地产领域若干历史遗留问题。深入开展“查尽责、查隐患、查投入，保安全”专项行动，举行省市联合突发环境事件应急演练，安全生产形势保持稳定。加大食品药品安全整治力度，保障群众饮食用药安全。加强大气污染防治，淘汰黄标车老旧车9971辆，秸秆禁烧实现“零火点”。我市运动员王键勇夺里约残奥会举重银牌。防震减灾工作获全省综合考评一等奖。档案、地方志等工作继续加强，民族宗教、妇女儿童、老龄等事业取得新成绩，气象、机关事务等工作取得新进展。

七是加强政府自身建设，治理能力不断提升。深入践行“三严三实”，扎实开展“两学一做”学习教育、“讲看齐、见行动”学习讨论和“淮北转型靠什么、我为崛起做什么”大讨论活动。我市首部地方性法规《淮北市城乡规划条例》正式施行。在全省率先建立“1+8+17+10”政府重大事项决策机制，决策科学化、民主化、法治化水平显著提升。“七五” 普法规划全面启动。自觉接受人大监督和政协监督，办理市人大代表议案2件、建议100件，市政协委员提案175件。市长热线、市长信箱办结率100%，获评全国网民留言办理工作先进单位。加大反腐倡廉力度，强化行政监察，推动经济责任审计全覆盖，惩治和预防腐败体系建设进一步加强。

回顾过去的一年，我们倍感欣慰的是，在困难和挑战面前，全市上下始终坚持发展为上，经济社会发展呈现十大亮点：一是荣获全国绿化模范城市、全国双拥模范城称号，文明城市创建再添重要砝码；二是在合肥成功举办《中国碳谷·绿金淮北国家创新驱动试验区发展报告》征求意见会和陶铝新材料产业发展座谈会；三是平山电厂二期、相邦陶铝新材料、万达城市综合体等一批重大项目落地，结束了我市产业“有山无峰”“缺大少强”的历史；四是民生领域投入财政资金累计达117.5亿元，为历年最多，群众拥有实实在在的获得感；五是全市工业企业利税、利润增速双升至全省第2位；六是濉溪铝基高端金属材料产业集聚发展基地获批第二批省级战略性新兴产业集聚发展基地；七是服务业对经济增长的贡献率达51.4%；八是棚改获批资金额度、开工建设率、基本建成率均居全省第一，投资力度、推进速度、工作透明度前所未有；九是建成五大利企便民服务中心，“房产超市”经验在全省复制、全国推广；十是成功举办第二届“中国创翼”青年创业创新大赛“互联网+”单项赛全国总决赛。

回顾过去的一年，我们倍受鼓舞的是，市第八次党代会描绘了淮北未来五年的发展蓝图，确立了闯出转型崛起的“七条新路”。在历届市委、市政府艰辛探索的基础上，我们紧紧围绕习近平总书记关于“绿水青山就是金山银山”的重要论述，借助国家智库力量，高起点谋划并推动争创中国碳谷·绿金淮北国家创新驱动试验区战略，寻求破解难题的“金钥匙”、思考医治“矿竭城衰”的“良药方”，集聚转型升级的“全要素”，建设政治生态、经济生态、自然生态“三位一体”的绿金样板城市，得到省委、省政府充分肯定和大力支持，全市形成广泛共识，为我们在新形势谋求新发展、新起点创造新业绩增添了强大动力。

回顾过去的一年，我们倍加珍惜的是，全市经济社会发展成绩来之不易，这是省委、省政府和市委正确领导的结果，是全市人民团结奋斗的结果，是历届历任班子坚持不懈、奋发进取打下坚实基础的结果。在此，我代表市人民政府，向全市人民，向各民主党派、各人民团体和各界人士，向各位老领导、老同志，向驻淮解放军指战员、武警官兵和政法公安干警，向中央及省驻淮单位，向所有关心、支持、参与淮北发展的投资者、建设者，表示诚挚的感谢，并致以崇高的敬意！

我们也清醒地看到，在转型崛起的征程中，还存在不少问题和挑战。经济总量较小，实体经济困难较多，稳定经济发展和财政增长的压力大、任务重、挑战多；战略性新兴产业比重较低，现代服务业发展较慢，民营经济发展不足；城乡发展不够均衡，基础设施、社会建设和民生领域还有大量短板亟待补齐；少数干部观念落后，担当不足，创新不够，效能建设、作风建设、廉政建设需要进一步深化。对此，我们一定高度重视，采取有力措施，切实加以解决。

2017年：奋发有为，全面加快转型崛起步伐

今年（2017年）是实施“十三五”规划的重要一年，是供给侧结构性改革的深化之年，也是全面贯彻落实市第八次党代会决策部署的关键一年，做好今年（2017年）工作，意义重大。国家促进中部地区崛起，省委、省政府高度重视皖北振兴发展，我市作为国家资源枯竭城市转型试点、中小城市综合改革试点和智慧城市试点，各种政策机遇叠加，工业经济止跌企稳，企业效益持续提高，转型升级步伐加快，发展动能逐步释放，发展环境持续优化。总体判断，机遇大于挑战，担当必有作为。我们必须进一步强化目标导向、问题导向和效果导向，进一步强化机遇意识、进取意识、责任意识，进一步强化战略思维、创新思维、底线思维，奔着问题去，迎着困难上，敢于担当，善做善成，奋力把淮北转型发展推向新的水平。

政府工作的总体要求：全面贯彻党的十八大、十八届三中、四中、五中、六中全会和习近平总书记系列重要讲话特别是视察安徽重要讲话精神，深入贯彻市第八次党代会精神，全面落实创新、协调、绿色、开放、共享发展理念，坚持稳中求进工作总基调，以中国碳谷·绿金淮北战略为引领，以提高发展质量和效益为中心，以推进供给侧结构性改革为主线，坚持新型工业化、新型城镇化双轮驱动，促进经济社会持续平稳健康发展，为实现淮北转型崛起奠定坚实基础。

经济社会发展的主要预期目标：生产总值比上年增长7.5%左右；规模以上工业增加值增长7%；固定资产投资增长9%左右；财政收入增长3%；社会消费品零售总额增长10.5%；外贸进出口保持稳定；城镇、农村居民人均可支配收入分别增长7.5%、8%；城镇登记失业率控制在4.2%以内。节能减排完成年度目标任务。

主要做好九个方面的工作。

一、深化供给侧结构性改革，在推动经济提质增效上求突破

把稳定经济发展作为首要任务，统筹推进“去降补”，不断提升经济发展的质量和效益。

稳步推进煤炭去产能。按照中央和省部署，综合运用市场机制、经济手段和法治办法，协助煤炭企业去产能。指导企业细化职工分流安置方案，拓宽职工分流安置渠道，维护企业与职工合法权益。加强市企合作，合力推进企业“三供一业”分离移交工作，进一步改善矿区职工居住环境。

帮扶实体经济降成本。全面抓好降成本政策落实，在减税降费和降低人工、用能、用地等要素成本上加力增效，着力降低制度性交易成本。抓住市经济开发区新区列为国家第一批增量配电业务试点机遇，支持更多电力企业与园区及符合条件的企业开展电力直接交易。组织开展“送政策进企业”“干部帮联企业”等系列活动，用心用情用力为企业解决发展难题。

强化项目带动补短板。发挥重大项目对扩大有效投入，推动供给侧结构性改革的重要作用，全年续建、新开工和储备项目543个，总投资2859.7亿元，年计划完成投资514.3亿元。开工建设淮北至阜阳、蚌埠快速铁路等一批重大基础设施项目，淮萧客车联络线、符夹线扩能改造工程建成通车，淮水北调市级配水工程、南坪船闸及浍河航道工程竣工，补齐基础设施和生态环境等方面的突出短板。

持续扩大需求促消费。组织扩消费系列活动，稳定传统消费，培育网络消费、信用消费、服务消费等增长热点。完善商业网点规划，推动传统商圈提档升级，打造特色街区和社区商业街区。加快电子商务专业园区建设，实施濉溪县电子商务进农村综合示范县工程，争创一批省级电子商务示范园区。推动跨境电商突破发展。结合房地产去库存，优化供给结构，完善优惠政策，鼓励新市民和农民进城购房，促进房地产市场平稳健康发展。

二、深入实施工业强市战略，在加快产业升级上求突破

进一步突出工业经济主导地位，加快“调转促”步伐。以相邦陶铝新材料为龙头，强力推进开发区转型升级，着力打造濉溪铝基高端金属材料、新型煤化工合成材料两大基地，重点实施135万千瓦新型高效洁净低碳发电机组、华润金蟾药业中药配方颗粒制剂、华孚现代纺织服装产业园、易房新型墙体材料、完美食品、今麦郎食品、金龙AG玻璃、10万吨/年己内酯、弘邦天力、新能源车体等“十大工业项目”，为振兴实体经济提供强劲支撑。

大力实施创新驱动。围绕产业链，部署创新链，完善资金链，打造人才链，配套政策链。推动创新平台建设。实施开发园区科技提升计划，加快高新区、高新基地等创新载体建设，重点打造新型煤化工合成材料、铝基高端金属材料等产业研发平台，争创国家级铝制品检验检测中心，加快碳、陶铝新材料产业化进程。放大“源创客”“梧桐树”“方正智谷”集聚效应，发挥“江淮双创汇·走进淮北”带动作用，孵化创新型小微企业。加快创新主体培育。深入实施高新技术企业和“专精特新”中小企业培育计划，推动大中型工业企业研发机构全覆盖。新认定国家高新技术企业20家、省创新型（试点）企业3家、省级企业技术中心4家。实施“互联网+制造业”行动，培育“互联网+”制造业企业30家、智慧工厂20家。打造创新人才高地。实施“招才引智计划”，引进高层次创新创业团队，遴选3—5个创新团队进行重点支持。加强优秀企业家队伍建设，制定并实施“领军型企业家”“成长型知名企业家”“创新型企业家”培养计划。扎实推进“相城金蓝领”万人计划，推动产教融合和校企合作，弘扬工匠精神，培养壮大高技能人才队伍，加快建设技工大市。

优化工业产业结构。全年新增规模以上工业企业80家，工业投资增长8%。加快传统产业改造提升。实施产业升级和技术改造项目140个，开展增品种、提品质、创品牌“三品”专项行动，争创安徽名牌产品5件。加快成长产业做大做强。深入推进“百亿口子”行动计划，争取口子东山产业园年内主体工程完工，基本具备投产条件。依托安徽矿机、龙波电气等骨干企业，推进机械制造向环保设备、数控化装备等高端方向拓展。围绕煤焦化副产品、卓泰芳烃类产品及己内酯，推动下游项目建设，不断延伸产业链条。加快新兴产业创新发展。聚焦陶铝新材料推广应用、市场培育和产业塑造，打造以陶铝新材料为产业链核心的中国陶铝峰。加快发展新能源产业，实施两淮采煤沉陷区国家光伏领跑者项目。

大力发展现代服务业。培育城市商圈、大型专业市场，加快万达城市综合体建设，确保温哥华酒店投入运营，进一步提升城市服务功能。加快发展第三方物流和多方物流，确保中农批物流园年内建成，努力把传化公路港物流打造成为全国公路物流网络的重要节点。实施“快递下乡”工程，规划建设1—2个快递物流园。加快发展文化旅游产业，重点推进隋唐运河古镇、台湾文创园、口子文化博览园建设，打造一批精品景点、品牌景区。新增规模以上现代服务业企业15家、省级服务业集聚区1个以上。支持相山区、烈山区争创国家全域旅游示范区。

提升农业现代化水平。深入推进农业供给侧结构性改革，增加绿色优质农产品供给，狠抓农产品标准化生产、品牌创建、质量监管，确保粮食安全。建设100万亩专用品牌粮食、20万亩设施蔬菜、30万亩良种繁育基地，培育“三品一标”农产品60个、省级以上知名农产品品牌20个。发展生态循环农业，实施化肥和农药零增长行动，建设现代生态农业示范区。拓展农业产业链条，推进“互联网+农业”，促进一二三产融合发展。新增土地流转面积5万亩，培育市级示范家庭农场100个，农民合作社示范社100个，农业产业化重点龙头企业140家，农产品加工业产值达570亿元。

三、全面加快新型城镇化进程，在促进城乡统筹上求突破

坚持以人的城镇化为核心，以城乡基础设施建设为支撑，突出主城区、东部新城区、南部次中心、高铁新区、产业新城五大板块，强力推进棚户区改造、智慧城市、中湖景区、东湖景区、规划展示馆及档案馆、会展中心、全民健身中心、骨干路网、城市出入口景观提升、黑臭水体治理等“十大城建工程”，不断完善城市功能，提高城市品质。

健全城乡规划体系。认真落实《淮北市城乡规划条例》，强化“多规合一”理念，扎实做好城市总体规划修编和报批工作。突出城市功能定位，积极参与皖北城镇群规划建设，推进徐州都市圈协调发展规划。完善城乡控规体系，做好老城区控规维护，编制公共服务设施综合规划、养老服务设施专项规划，提高城市综合服务水平。

优化城市发展空间。围绕“一主一次、组团发展”城市布局，促进各组团与主城区融合发展。组织实施一批利在当前、事关长远的重大基础设施项目，总投资200亿元，年计划完成投资80亿元，完善东部新城区功能，开工建设市人民医院东院区、市一中东校区，加快推进市职教园区二期。全面拉开城市框架，完成东部新城区路网、高铁新区配套设施、烈山和杜集污水管网等40个续建项目，开工建设人民西路、龙山北路、S101合相路二期、S254古毛路二期、S203煤化工快速通道二期和S337西临路，打开城市外向发展通道。优化骨干路网布局，加快推进古城东路、泉山北路立交桥、桓谭东路等项目，构建城市交通“微循环”。实施民生路东段、梅苑路、桂苑路等道路贯通工程，着力打通“断头路”“瓶颈路”，解决群众“出行难”。抢抓政策机遇，加快推进棚户区改造步伐，提高货币化安置比重，完成棚改项目81个、40526套，基本建成棚改安置房1.5万套，让群众成为棚改的最大受益者。

提升城市管理水平。深入开展全国文明城市创建活动，落细落实各项任务，力争2017年进入全国文明城市行列。深入推进新型智慧城市建设，建成智慧城管、平安城市二期、智慧教育、智慧交通、无线城市等一批项目，提高城市治理能力。加快推进生活垃圾卫生填埋场等环卫基础设施建设，实现全市农村生活垃圾治理全覆盖，城市生活垃圾分类试点提标扩面。实施公共自行车三期工程。完善物业管理办法，进一步提升物业管理水平。利用电厂余热资源，积极创造条件，争取启动实施城市供热工程。

加快建设美丽乡村。扎实推进镇政府驻地建成区和中心村规划建设，加强自然村环境整治，构筑农村基础设施体系。实施农村饮水安全巩固提升工程，重点解决5.8万人饮水安全问题。高水平规划建设榴园石榴小镇、段园葡萄小镇、渠沟芳香小镇等一批产业基础好、文化积淀深、生态环境美的特色小镇。争取临涣镇入选国家历史文化名镇。

四、突出抓好招商带动，在提升开放水平上求突破

始终把招商引资作为转型崛起的“生命线”，持之以恒推动大招商、招大商，以增量带动存量优化、总量扩张、质量提升，全年引进5亿元以上项目25个。

深入推进精准招商。聚焦中国碳谷·绿金淮北战略，坚持把招大引强作为重点，紧盯全球500强、全国500强、行业百强企业，有针对性地开展与央企和知名民企的对口招商，积极承接上海产业转移，在深圳、东莞等地组织专题招商活动，深化与台商交流合作。坚持把产业招商作为关键，围绕先进制造业和战略性新兴产业，科学规划产业路径和招商路线，引进一批符合国家产业政策、贴近转型实际的新业态、新模式。坚持招商引资与招才引智、招园引制相结合，加快建设中德专利产业园。

提升外贸发展水平。主动融入“一带一路”战略，深化对外交流合作，高水平引进来，大规模走出去。加强外贸平台建设，建设青龙山铁路无水港二期，提升海聚科技公用型保税仓库运营能力，推动“进口直通、出口直放”。组织企业参加广交会、华交会等国内外知名会展，加强出口品牌建设，完善跨区域合作机制，开拓国际国内市场。加大新备案登记企业帮扶力度，引导帮助中小企业开展进出口业务。

优化投资发展环境。完善园区基础设施和生产生活性服务设施，加快市经济开发区新区标准化厂房、新型煤化工合成材料基地燃气设施等工程建设，提升配套能力。创新租赁、兼并、改建、嫁接等形式，盘活园区闲置低效土地和工业闲置资产，为园区和企业转型升级注入新活力。优化招商服务，实行招商项目行政审批全程代办、涉企服务全力帮办，打造服务企业“升级版”。扎实做好创建国家社会信用体系建设示范城市工作，开展特色诚信创建活动，营造良好信用环境。

五、持续深化重点改革，在激发动力活力上求突破

用足用好国家中小城市综合改革试点等政策机遇，在推动重点领域和关键环节改革上持续发力、务求实效。

深化行政管理体制改革。动态完善权责清单、涉企收费清单，实施全方位公共服务清单和全口径行政权力中介服务清单，建成市县镇三级公共服务清单和市县两级行政权力中介服务清单。强化事中事后监管措施，全面推行“双随机、一公开”监管改革。深化商事制度改革，探索“多证合一”，激发市场主体活力。推广“互联网+政务服务”，开展全国政务“云平台”应用试点，让基层群众办事更方便、更高效、更舒心。

深化财政金融体制改革。完善财政体制机制，科学划分事权财权，理顺市县（区）财政分配关系。推行全面规范、公开透明的预算制度。深化国资国企改革，以管资本为主推进国有资产监管机构职能转变，确保国有资产保值增值。加强政府债务管控，全面提升PPP模式运营管理水平。成立市金融控股集团，探索成立市工业发展投资公司，发挥市扶持产业发展专项资金、产业投资基金的引导作用，全面提升服务实体经济能力。发挥皖北融资中心平台优势，对接多层次资本市场，推动企业挂牌上市。

深化园区管理体制改革。坚持把园区作为转型发展的主阵地，支持各开发园区发挥政策优势，突出主导产业，实现错位发展。以产城融合的新理念，积极探索“区政合一”，着力破除体制机制障碍。借鉴外地先进经验，按照产业新城建设模式，引进有成功经验的知名企业，参与园区投资、建设、招商和运营，规划建设碳谷产业园、陶铝产业园，激发园区发展活力。

深化农村综合改革。实施农村土地所有权、承包权、经营权分置改革，巩固农村土地确权登记颁证成果。开展农村集体资产股份合作制改革，推动资源变资产、资金变股金、农民变股东改革试点。深化供销社改革，推进国有农场改革。大力发展农村普惠金融。全面推开农业三项补贴改革，让农民群众吃上“定心丸”。

大力发展非公经济。发挥民营经济主力军作用，像抓招商引资一样抓民营经济，开展“温商暖企”行动，提振企业发展信心，扶持培育龙头企业和成长型中小企业，为企业家创新创业“清障搭台”“保驾护航”。实施市场主体增量工程，激发全民创新创业热情，新增私营企业5000户、个体工商户1.2万户。积极推动濉溪国家级农民工返乡创业试点建设，支持更多的“草根”脱颖而出，成功实现“小目标”。

六、加强生态城市建设，在厚植绿色优势上求突破

坚持生态优先，绿色发展，全面贯彻海绵城市、低碳城市发展理念，进一步保护和改善环境，建设生态文明示范区，争创国家森林城市、国家环保模范城。

注重生态保护修复。继续实施石质山场绿化提升、森林长廊、生态廊道、农田林网建设工程，推进花山公园、儿童公园、龙岱河景观带、凤凰山隧道游园等项目建设，申报中湖国家湿地公园、相山国家森林公园。加强耕地资源保护，完成永久基本农田划定工作，推进山水林田湖综合整治。积极开展重点用水企业地下水置换工作。加强城市生态修复，实施三五山及烈山、卧牛山山体修复等工程，扮靓城市门户。

加大环境治理力度。加强“三线三边”环境综合治理，全力打好大气、水、土壤污染防治三大战役。加强水泥、电力企业粉尘排放提标改造，淘汰黄标车和各类燃煤小锅炉，确保PM10年均浓度保持在73微克/立方米以内。加强河道整治，建立“河长制”，实施老濉河水生态环境综合整治工程，尽快改变脏乱差，改善水环境。加强农业面源污染防治，推进固体废弃物减量化、无害化处置。

提高资源利用效率。严格环境准入，实行能源和水资源消耗、建设用地等总量和强度双控制度。开展煤炭清洁高效利用等“四大专项行动”，推进重点行业绿色节能改造。实施绿色交通工程，推广应用新能源公交车，提高城市公共交通分担率。推进秸秆综合利用产业化，加快浦发生物质发电、上海电气生物质能热电项目建设，农作物秸秆综合利用率达85%。

七、坚持共建共享发展，在增进民生福祉上求突破

始终把保障和改善民生放在突出位置，落实共享发展理念，继续组织实施好省市各项民生工程，使发展成果更多、更公平惠及全市人民。

加快脱贫攻坚步伐。严格按照脱贫目标，建立全覆盖的精准扶贫、监测评估、防范返贫、责任落实四项机制，扎实推进精准扶贫、精准脱贫。深入实施脱贫攻坚十大工程，提高贫困群众脱贫致富能力，改善贫困村生产生活条件。完善稳定脱贫政策措施，细化压实工作责任，确保15个贫困村出列、8981人脱贫。

优先发展教育事业。深化教育综合改革，全面下放义务教育阶段学校管理权限。认真落实新建小区配套建设教育设施政策。实施第三期学前教育行动计划。深化职业教育市级统筹和办学模式改革，依托淮北职业技术学院、淮北技师学院组建职业教育联盟，搭建职教人才成长“立交桥”。实施教师陪伴成长、全员全程育人工程。加大教师培训和校干培养力度，优化教师资源结构和配置，全面推行中小学教师“无校籍管理”“县管校聘”制度。

推动就业和社会保障工作。实施就业优先战略，统筹做好高校毕业生、农村转移劳动力等重点群体就业，妥善做好化解过剩产能中的职工分流安置工作。织密扎牢民生保障网，全面实施“全民参保计划”，加快机关事业单位工作人员养老保险制度改革，基本建立统一的城乡居民医疗保险制度。

加快健康淮北建设。稳妥有序实施全面两孩政策，完善卫生计生融合服务体系。深化医药卫生体制综合改革，加强医疗、医保、医药“三医联动”。开展医养结合示范项目建设，推进城乡居家养老服务中心建设，逐步实现城镇居家养老服务全覆盖。加强全民健康教育和应急救护知识普及教育。落实全民健身国家战略，推动全民健身和全民健康深度融合。

完善公共文化服务体系。加强社会主义核心价值观建设，提高市民文明素质和城市文明程度。深入推进文化惠民工程，提升城乡基本公共文化服务标准化、均等化水平。加强文化遗产保护和利用工作，谋划打造“中国古乐之乡”，促进文化事业与旅游、科技、金融等产业融合发展。举办市第十届运动会暨第四届职工运动会。

统筹发展社会事业。完成第三次全国农业普查。大力发展社会福利和慈善公益事业。完善残疾人民生和权益保障机制。支持工会、共青团、妇联等群团组织开展好各项活动，统筹抓好外事侨务、民族宗教、编制、档案、保密、防空防震、科协、老干部、地方志、红十字会等工作。

八、着力创新社会治理，在维护社会和谐稳定上求突破

牢固树立发展决不能以牺牲安全为代价的红线意识，夯实基层基础工作，切实保障人民群众生命财产安全，争创全国社会治安综合治理优秀城市。

深化平安淮北建设。构建立体化、数字化的社会治安防控体系，依法严厉打击各类违法犯罪活动。全面落实依法分类信访处理机制，发挥网上信访主渠道作用，提升基层综治维稳信访工作中心（站）效能，着力解决群众合理合法诉求。加强互联网安全管理，提高依法管网和网络突发事件处置能力，防范和打击新型网络诈骗。强化应急管理体系建设，进一步完善监测预警和隐患排查机制。

强化安全生产和食品药品安全监管。深入推进安全生产领域改革发展，严格落实安全生产责任制，构建安全风险分级管控和隐患排查治理双重预防机制。深化“铸安”行动，推动隐患整改全覆盖，坚决遏制较大以上安全事故。加强食品安全管理，健全食品药品安全治理体系，争创省级食品药品安全城市。

推动军民深度融合发展。巩固全国双拥模范城创建成果，持续开展“双拥在基层”和“四方共建”活动，进一步密切军地军民关系，促进军政军民大团结。

九、坚持从严从实要求，在强化责任担当上求突破

为淮北人民谋利益、谋幸福是政府的首要职责。新形势下必须以新理念统领政府工作，以新要求加强自身建设，全面提高为民服务本领。

始终做到忠诚履职。巩固“两学一做”学习教育成果，认真开展“讲看齐、见行动”学习讨论，树牢“四个意识”特别是核心意识、看齐意识，坚决维护党中央权威和集中统一领导，坚决维护习近平总书记党的领导核心地位。坚定理想信念，强化理论武装，增强中国特色社会主义道路自信、理论自信、制度自信、文化自信，让信仰永驻于心，居之无倦，行之以忠，转化为推动淮北发展、造福全市人民的强大动力。

始终做到依法行政。深入实施“七五”普法规划，加快法治政府建设。健全依法决策机制，实现重大事项合法性审查机制全覆盖。扎实推进行政执法体制改革，推进相对集中行政处罚权和综合执法。强化政务公开，推进网络问政。自觉接受人大监督、政协监督、司法监督、社会监督，认真办理人大代表议案、建议和政协委员提案，广泛听取各民主党派、工商联、无党派人士意见，让行政权力始终在法治轨道上运行，让人民群众更有效地监督政府。

始终做到清正廉洁。严格落实全面从严治党要求，规范党内政治生活，加强和接受党内监督，强化权力制约监督和风险防控，把权力关进制度的笼子，套上“紧箍咒”、筑牢“防火墙”。牢固树立过“紧日子”的思想，严控“三公”经费支出，推动落实中央八项规定精神和省市相关规定常态化、长效化。加强行政监察，用好审计利器，坚决整治和查处侵害群众利益不正之风和腐败问题。严格执行党风廉政建设各项规定，全面落实“两个责任”，打造政治生态上的绿水青山。

始终做到勇于担当。坚持守土有责、守土尽责，直面改革发展过程中的关键矛盾，以改革破解难题，以创新推动发展，以担当成就事业，用“辛苦指数”换群众“幸福指数”、添城市“美丽指数”。持续加强效能建设，坚决纠正不作为、慢作为、乱作为突出问题。把抓落实作为重大政治责任，盯住重点任务，打通政策落实“中梗阻”“最先一公里”“最后一公里”，确保各项决策部署落地生根、开花结果。

各位代表！寒凝大地发春华，不忘初心谱新篇。新的一年，让我们在中共淮北市委的坚强领导下，凝心聚力，狠抓落实，行稳致远，为我市闯出一条转型崛起新路而努力奋斗！以优异成绩迎接党的十九大胜利召开！
